# Beneficial impacts of a national smokefree environments law on an indigenous population: a multifaceted evaluation

**DOI:** 10.1186/1475-9276-8-12

**Published:** 2009-04-30

**Authors:** Richard Edwards, Heather Gifford, Andrew Waa, Marewa Glover, George Thomson, Nick Wilson

**Affiliations:** 1 Health Promotion and Policy Research Unit, Department of Public Health, University of Otago, Wellington, New Zealand; 2 Whakauae Research Services, Whanganui, New Zealand; 3 Health Sponsorship Council Research and Evaluation Unit, Wellington, New Zealand; 4 Auckland Tobacco Control Research Centre, School of Population Health, University of Auckland, Auckland, New Zealand

## Abstract

**Background:**

Smokefree environments legislation is increasingly being implemented around the world. Evaluations largely find that the legislation is popular, compliance is high and report improved air quality and reduced exposure to secondhand smoke (SHS). The impact of the legislation on disadvantaged groups, including indigenous peoples has not been explored. We present findings from a multifaceted evaluation of the impact of the smokefree workplace provisions of the New Zealand Smokefree Environments Amendment Act on Māori people in New Zealand. Māori are the indigenous people of New Zealand. The Smokefree Environments Amendment Act extended existing smokefree legislation to almost all indoor workplaces in December 2004 (including restaurants and pubs/bars).

**Methods:**

Review of existing data and commissioned studies to identify evidence for the evaluation of the new legislation: including attitudes and support for the legislation; stakeholders views about the Act and the implementation process; impact on SHS exposure in workplaces and other settings; and impact on smoking-related behaviours.

**Results:**

Support for the legislation was strong among Māori and reached 90% for smokefree restaurants and 84% for smokefree bars by 2006. Māori stakeholders interviewed were mostly supportive of the way the legislation had been introduced. Reported exposure to SHS in workplaces decreased similarly in Māori and non-Māori with 27% of employed adult Māori reporting SHS exposure indoors at work during the previous week in 2003 and 9% in 2006. Exposure to SHS in the home declined, and may have decreased more in Māori households containing one or more smokers. For example, the proportion of 14–15 year old Māori children reporting that smoking occurred in their home fell from 47% in 2001 to 37% in 2007. Similar reductions in socially-cued smoking occurred among Māori and non-Māori. Evidence for the effect on smoking prevalence was mixed. Māori responded to the new law with increased calls to the national Quitline service.

**Conclusion:**

The New Zealand Smokefree Environments Amendment Act had a range of positive effects, including reducing SHS exposure among Māori communities. If the experience is replicated in other countries with indigenous populations, it suggests that comprehensive smokefree environments legislation will have beneficial effects on the health of indigenous groups and could contribute to reducing inequalities in health within societies.

## Introduction

Smokefree environments legislation is increasingly being implemented around the world. Evaluations of the impact of such legislation have mostly been highly positive [1]. Existing evaluations have generally focused on the broad impact of the legislation on populations and societies, as well as more specific evaluations focused on exposure to secondhand smoke (SHS), and health and economic effects in key sectors such as the hospitality industry. We are not aware of any evaluations of smokefree legislation that have examined the effect on particular sectors of society most affected by the tobacco epidemic such as indigenous peoples.

The New Zealand Smoke-free Environments Amendment Act (SEAA) [2] became law on December 10 2003. The SEAA strengthened existing legislation by introducing a range of tobacco control measures including restricting the display and sales of tobacco products, reducing under 18-year-old access to tobacco products, and providing for stronger future regulation of smoking product information and warnings. The SEAA stipulated that the buildings and grounds of all schools and early childhood centres should be totally smokefree from January 1 2004 and nearly all indoor workplaces from December 10 2004. This included bars, casinos, members' clubs and restaurants; and non-office workplaces. Several partial exemptions were allowed, notably prisons, hotel and motel rooms, and residential establishments such as long-term care institutions and rest homes. Traditional Māori indoor settings such as marae (traditional Māori community meeting spaces) were only included in the legislation where they were defined as a workplace.

Pre-existing restrictions introduced a decade earlier in the 1990 Smoke-free Environments Act [3] banned smoking in many indoor workplaces, including in most shared offices, and introduced partial restrictions (≥ half of the area non-smoking) for work cafeterias, restaurants and meal-serving areas of pubs and other licensed venues. The overall impact of the 2003 SEAA has been evaluated and is described elsewhere [4].

Smoking is a major public health problem in New Zealand. Prevalence is high among young people and is particularly strongly associated with lower socio-economic position and Māori ethnicity [5]. Māori, the indigenous people of New Zealand, make up 15.3% of the New Zealand population [6]. Whilst adult smoking prevalence in New Zealand was 24.1% in men and 22.9% in women in 2006, prevalence among Māori was 40.7% among men and 50.6% among women [7]. Smoking prevalence among Māori women is among the highest in the world for any female population group. Due to this high smoking prevalence, tobacco use has a particularly adverse impact on the health of Māori communities [8], and smoking contributes to the large disparities in health between Māori and non-Māori in New Zealand [9,10].

Health data that indicate smoking-related conditions impact disproportionately among Māori: Māori have a higher incidence of lung cancer and other tobacco-related cancers, sudden infant death syndrome, pregnancy related complications and respiratory conditions, especially in children. Smoking is also likely to be a major reason for the higher rates of heart disease, respiratory infections and conditions such as asthma, otitis media, and the adverse outcomes of diabetes seen among Māori [11].

The inequalities in tobacco smoking and the health-related harms it causes are of great concern to Māori. They are also a concern for the government and the health sector; for ethical reasons of ensuring social justice, and because the New Zealand Government is committed to improving Māori health under the obligations of the Treaty of Waitangi. This treaty was signed in 1840 between representatives of the British Crown and Māori chiefs. It is generally regarded as the founding agreement between the Crown and Māori in the modern state of New Zealand [12]. In particular, Article three of the Treaty translates into an obligation for Crown agencies to work to ensure that Māori citizens enjoy the same rights as others, including the right to good health.

The 1988 Royal Commission on Social Policy [13] advocated three principles and provisions which could be derived from the Treaty of Waitangi and applied to Māori health issues. These are reiterated in He Korowai Oranga – the government's Māori health strategy [11]. The principles are protection, partnership and participation. Protection refers to the Crown's obligation to proactively seek opportunities to advance Māori health [12]. Partnership refers to working together with Māori communities to develop strategies for Māori health gain [11], and participation in this context refers to developing a full Treaty-based decision making process by and for Māori in the development, governance and implementation of tobacco control strategies [12].

An additional dimension to tobacco control among Māori communities is that tobacco use can be seen as a part of the colonisation process. Prior to European contact there was no smoking or other use of tobacco by Māori [14]. Tobacco was introduced to New Zealand by early Pākehā (non-Māori colonists of largely European origin) explorers and traders and was used as a currency and article of trade. Its use was widely adopted among Māori, and the New Zealand Government acknowledged the heavy involvement of Māori with tobacco as early as 1894 [14].

Colonisation initiated a catastrophic decline in the Māori population due to the impact of wars, appropriation of land and resulting economic hardship, and European introduced diseases and health damaging products such as alcohol and tobacco [15]. By 1900 Māori public health leaders recognised the adverse effect smoking was having on Māori health [14].

Over at least the last hundred years Māori have implemented a range of initiatives to counteract the impacts of colonisation on the health of their population. In particular, political activities in the 1970s resulted in increased acknowledgment of a separate Māori identity and increased recognition of rights needing to be addressed as a result of breaches to the Treaty of Waitangi by the Crown. Examples of these activities included the 1972 Māori Language Petition, amendment of the Māori Affairs Amendment Act 1974, the 1975 Māori Land March, the Treaty of Waitangi Act 1975 establishing the Waitangi Tribunal, tribe-led protests and land occupations in 1977 at Bastion Point and in 1978 at Raglan [16].

More recently tobacco has been positioned as a tool of colonisation [17] and many Māori are now calling for more substantive approaches to tobacco control including; denormalisation campaigns, exposing the role of the tobacco companies in recruitment of Māori smokers, using economic modelling to discuss the role of tobacco in poverty and even total bans on tobacco sales [18]. This call has been picked up by some Māori political leaders. Many of the more cutting-edge proposals for tobacco control in New Zealand are advocated by Māori leaders. For example, the original bill to review the 1990 Smokefree Environments Act, the Smoke-free Environments (Enhanced Protection) Amendment Bill, was introduced by the Māori Member of Parliament (MP) Tukuroirangi Morgan in July 1999, with support later from Tuariki Delamere MP.

The SEAA was introduced in December 2003. This followed sustained advocacy efforts, based on evidence of growing public support and also persisting significant workplace SHS exposures, in particular of greater exposure among blue collar workers and Māori [19-21].

The main objective of the SEAA was identified as being to reduce SHS exposure by extending protection to workers still exposed to SHS in indoor workplaces after the 1990 Smoke-free Environments Act. Guiding principles identified from New Zealand Ministry of Health strategy documents [11,18,22-24] and the Parliamentary Health Committee report [25] into the legislation were, firstly, that the SEAA should promote equity in health by improving health among groups disproportionately affected by tobacco smoking and SHS exposure, such as Māori. Secondly, policies should be congruent with the principles and provisions of New Zealand's founding agreement between the Crown and Māori, the Treaty of Waitangi.

An evaluation of the SEAA from a Māori perspective is therefore particularly relevant and important. This paper reports the key findings of a multi-faceted evaluation of the smokefree provisions (excluding the impact of the introduction of smokefree schools and early learning establishments) of the SEAA, focusing particularly on the impact on Māori people. Our main aim was to evaluate the degree to which the SEAA achieved its primary objective of reducing SHS exposure in the workplace among Māori.

## Methods

### Evaluation methods

The methods of the evaluation have been described more fully in a published report [26] and summary paper [4]. We acknowledge that our method adapted methods from the existing evaluation literature and was not a kaupapa Māori approach. Kaupapa Māori research has been defined as research that is culturally safe, relevant and appropriate, and is controlled by Māori, for Māori and with Māori. It addresses Māori needs and gives full recognition to Māori culture and value systems, upholds the interest and mana (dignity and respect) of the group, and embraces traditional beliefs and ethics which incorporate resistance strategies that embody the drive for tino rangatiratanga (unqualified indigenous authority and self-determination) [27]. Such an approach may, for example, have focused more on the degree to which the legislation promoted Māori aspirations and participation.

We developed a logic model adapted from the evaluation model of the US Centers for Disease Control [28], and the logic model used for the evaluation of the Scottish smokefree legislation [29] for the evaluation [26]. This incorporated process, and core (direct, anticipated impacts relating to primary objectives) and non-core (indirect, possible outcomes relating to secondary objectives) outcome indicators. The process and outcome information areas used to structure the evaluation were as follows:

#### Process indicators

➢ Public and stakeholder knowledge, attitudes and support for smokefree policies

➢ Participation, dissemination of information, enforcement activities and compliance monitoring

#### Core outcome indicators

➢ Exposure to SHS in the workplace (principal outcome measure)

➢ Health impacts attributable to SHS exposure

#### Non-core outcome indicators

➢ Exposure to SHS in public places and private places such as homes

➢ Smoking prevalence and smoking-related behaviours

➢ Economic impacts

Information on the process of monitoring and compliance and on health and economic impacts were included in the evaluation, but were not available by ethnicity or socio-economic position, so are not described further in this paper.

### Information sources

For each of the process and outcome indicators, we identified data sources from literature searches, and from consulting members of the project team and other key informants. Data sources included existing papers, reports, policy documents, national health and demographic statistics. We summarised the evidence from each relevant data source. Where the data or existing analyses were judged insufficient for the purposes of the evaluation, and where practicable, we carried out additional analyses or studies. Additional studies and analyses reported in this paper are: further analysis of evaluation data from the Health Sponsorship Council (HSC) Monitor Surveys, an exploration of the experiences and attitudes of Māori stakeholders to the introduction and implementation of the SEAA, and an analysis of national Quitline data. The data presented is therefore a combination of summaries of relevant data and analyses carried out by other researchers, and key findings of additional quantitative analyses of existing data sources and additional qualitative research carried out by the authors.

Evidence on public and stakeholder knowledge, attitudes and support for smokefree policies; exposure to SHS in the workplace and at home; and the frequency of socially-cued smoking reported by smokers in pubs and bars, restaurant and nightclubs came from the HSC Monitor Surveys. Additional data came from the ASH Year 10 children's survey (for exposure to SHS in the home), and from National Research Bureau surveys, 1989–2001, for pre SEAA data on exposure to SHS in the workplace and home.

The HSC Monitor Surveys (2003–6) were nationally representative surveys with 2000–2500 participants aged 15 years or above, with a boosted Māori population sample of about 900 (500 in 2003). The surveys included questions that were developed specifically to monitor the SEAA. [30] Data collection for 2003–4 occurred prior to implementation of the main smokefree provisions of the SEAA and for 2005–6 after implementation. A general population sample was obtained through random digit dialling in 2003 and 2004, and by random sampling of private household numbers in 2005 and 2006. The person with the next birthday present at the time of the call (2003 or 2004) or within the household (2005 and 2006) was invited to participate. The Māori sample was made up of Māori participants from the general sample and from an additional random sample of households of individuals who self-identified as Māori on electoral rolls. Participants were interviewed using Computer Assisted Telephone Interviews which included questions on many topics related to smoking, SHS exposure and the SEAA, with many questions common to several or all of the surveys.

Response was defined as the estimated proportion of eligible people contacted who agreed to participate (consent rate) and the proportion of the eligible people identified for inclusion who agreed to participate (response rate). During 2003–6 (2006 figures are provisional) in the general survey these ranged between 29–38% for consent rates (38% 2003, 35% 2004, 29% 2005, and 34% 2006) and 29–38% for response rates (44% 2003, 41% 2004, 32% 2005, and 38% 2006). For the Māori sample, the range was 39–79% for consent (79% 2003, 69% 2004, 47% 2005, and 39% 2006) and 35–63% (62% 2003, 63% 2004, 39% 2005, and 35% 2006) for the response rate. There were no obvious trends in response and consent rates over time, except that the Māori over-sample consent and response rates were much lower in 2005–6 than 2003–4, possibly due to changed reporting practices between survey companies.

We carried out additional analyses of the 2003–2006 HSC Monitor Surveys, weighting the findings to the age, sex and ethnicity (proportion of Māori and non-Māori) distributions of the 2001 census [26]. This ensured that changes in the distribution of survey responses were not the result of changes in the age, gender and ethnicity distribution of the populations participating in the surveys. Results were stratified by gender, ethnicity (Māori/non-Māori), smoking status (smoker/non-smoker) and by level of household income.

The ASH Year 10 Smoking survey is a national cross-sectional questionnaire survey of 14–15 year old children [31]. Data are collected anonymously using self-completion questionnaires which have included questions on children's reports of parental smoking and SHS exposure in the home since 2001. Between 1999 and 2007 an average of over 30,000 students participated each year, representing more than 70% of all eligible schools and around half of all Year 10 students in New Zealand.

The National Research Bureau (NRB) studies were conducted in 1989, 1991, 1996 and 2001. [19,21,32,33] The surveys recruited a demographically representative sample of adults aged 15 years and over from households included in regional telephone directories, and collected data using telephone interviews. Response rates in the NRB surveys were around 60–70%, varying with the methods used to define non-responders. The interviews included questions on self-reported SHS exposure in the workplace. The 1989, 1991 and 1996 surveys also included questions on SHS exposures in the home and details on the workplace smoking policy. They therefore provided information on baseline data and trends in SHS exposure at work and in the home prior to the SEAA implementation.

The New Zealand Tobacco Use Survey was first carried out in 2006, and includes data on a wide range of topics including SHS exposure in the home and at work collected through face-to-face interviews [7]. A complex systematic stratified sampling scheme was used to achieve a nationally representative sample of adults aged 15–64 years with over-sampling of Pacific Islanders, Māori and 15–24'year olds. The sample size was 5703 (response rate of 75%), of whom 1071 were Māori.

The New Zealand Health Survey was carried out using face-to-face interviews in 1996/7, 2002/3, and 2006/7. A systematic stratified sampling scheme was used to achieve a nationally representative sample of adults aged 15 years and over, with over-sampling of some ethnic groups including Māori. In the 2006/7 survey 12488 adults, including 3160 Māori. Response rates were 72% in 2002/3 and 68% in 2006/7 [34].

Evidence about the experiences and attitudes of Māori stakeholders to the introduction and implementation of the SEAA came from eight in-depth interviews, six of which were conducted as face-to-face interviews by a Māori interviewer; and two by telephone interview by a research consultancy company. The Māori stakeholders were selected purposively to get a cross-section of opinions and experiences, and comprised four interviewees from Māori non-governmental organisations, a public health manager, a smoking cessation worker, a union official and a bar manager [26]. The eight interviews with Māori stakeholders were part of a larger qualitative study involving 34 interviews which aimed to document enforcement activities, and investigate the attitudes, beliefs, experiences and perceptions of stakeholders about the process of implementation, enforcement and compliance with the smokefree aspects of the SEAA [26]. The interviews with Māori stakeholders focused particularly on participants' hopes, aspirations and beliefs regarding the changes in legislation, their experiences as someone involved in implementing the changes and what they viewed as the wider impacts of the changes including the acceptability to Māori communities. We used content analysis to identify the main themes and illustrative quotes.

The main sources of information on quitting behaviour by ethnicity came from the monthly number of caller registrations and subsidised nicotine replacement therapy (NRT) vouchers issued by the national Quitline [35].

## Results

### Attitudes among Māori towards secondhand smoke and support for smokefree policies

We found evidence of strong and growing support for the new smokefree legislation and its underlying principles among Māori and non-Māori (table 1). For example, data from the HSC surveys showed that the social unacceptability of smoking around children was high among Māori and non-Māori before and after implementation of the SEAA and was 96.2% (95% CI 94.7% to 97.2%) among Māori and 96.6% among non-Māori (95% CI 95.5% to 97.4%) in 2006. In the 2004–2006 surveys, respondents were asked whether they thought that the dangers of SHS have been exaggerated. The proportion disagreeing increased from 59.0% (95% CI 55.7% to 62.3%) to 69.9% (95% CI 66.1% to 72.0%) among Māori respondents and from 63.1% (95% CI 60.5% to 65.7%) to 71.7% (95% CI 69.2% to 74.4%) among non-Māori between 2004 and 2006. In the 2006 Tobacco Use Survey, Māori respondents were less likely (27.5%, 95% CI 23.4% to 31.5%) than non-Māori (40.1%, 95% CI 38.4% to 41.8%) to state they were bothered a lot by others smoking around them and to believe that SHS is harmful. However, these differences may be confounded by a greater proportion of smokers among Māori, as smokers are much less likely to be bothered by SHS and believe SHS causes harm. [7]

**Table 1:** Support for rights of non-smokers and support for 2003 Smokefree Environments Amendment Act provisions in 2003–6 HSC Monitor Surveys (weighted to 2001 census)

	Year	All % (95% CI)	Māori **% **(95% CI)	non-Māori **% **(95% CI)
Right to live in a smokefree environment	2003	87.1 (85.4 to 88.6)	75.1 (70.1 to 79.6)	89.9 (88.1 to 91.4)
	2004	85.5 (83.7 to 87.1)	79.1 (74.3 to 83.2)	86.9 (85.0 to 88.6)
	2005	91.6 (90.2 to 92.9)	81.1 76.5 to 85.0)	94.1 (92.7 to 95.2)
	2006	91.9 (90.5 to 93.1)	82.0 (77.3 to 85.9)	93.9 92.5 to 95.1)

Right to work in a smokefree environment	2003	90.7 (89.3 to 92.0)	82.6 (78.0 to 86.4)	92.8 (91.3 to 94.0)
	2004	88.8 (87.2 to 90.2)	82.8 (78.4 to 86.5)	90.2 (88.5 to 91.7)
	2005	95.7 (94.6 to 96.6)	90.6 (87.1 to 93.2)	96.9 (95.9 to 97.7)
	2006	94.9 (93.8 to 95.9)	92.3 (88.7 to 94.8)	95.5 (94.2 to 96.5)

Right of bar and pub workers to work in a smokefree environment	2003	79.1 (77.1 to 81.0)	56.9 (51.4 to 62.2)	84.0 (82.0 to 85.9)
	2004	80.7 (78.7 to 82.5)	66.2 (60.9 to 71.2)	84.0 (81.9 to 85.9)
	2005	91.1 (89.7 to 92.4)	78.6 (73.8 to 82.6)	94.0 (92.6 to 95.2)
	2006	91.5 (90.1 to 92.8)	82.7 (77.9 to 86.6)	93.3 (91.9 to 94.5)

Right of restaurant workers to work in a smokefree environment	2003	84.4 (82.6 to 86.0)	67.8 (62.5 to 72.8)	88.3 (86.4 to 89.9)
	2004	85.3 (83.5 to 87.0)	77.2 (72.2 to 81.5)	87.2 (85.3 to 88.9)
	2005	94.3 (93.1 to 95.3)	88.3 (84.5 to 91.3)	95.7 (94.5 to 96.6)
	2006	95.6 (94.5 to 96.5)	93.4 (90.0 to 95.6)	96.0 (94.8 to 97.0)

Right of non-office workers to work in a smokefree environment	2003	89.4 (87.9 to 90.8)	80.7 (76.0 to 84.7)	91.4 (94.8 to 97.0)
	2004	88.3 (86.7 to 89.8)	81.7 (77.1 to 85.6)	89.8 (88.1 to 91.4)
	2005	95.2 (94.1 to 96.1)	90.8 (87.4 to 93.4)	96.2 (95.0 to 97.1)
	2006	94.7 (93.5 to 95.7)	89.8 (85.8 to 92.8)	95.7 (94.5 to 96.6)

**Key**: All %s refer to those expressing support defined as those who strongly agreed or agreed with each statement. Those who did not express support included people who disagreed or who were neutral or unsure.

Data from the HSC surveys showed that by 2006 there was overwhelming support (over 90% agreement, and 6% or less disagreement) for the right to live and work in a smokefree environment; and for indoor workers, including bar and restaurant workers, to work in a smokefree environment (table 1). Support among Māori respondents was slightly lower than non-Māori in 2006, but the absolute increase from levels of support in 2003 was greater among Māori than non-Māori. There were particularly large increases in support for the rights of hospitality workers to work in a smokefree environment among Māori between 2003 and 2006 (an absolute increase of 25–26%, compared with 8–9% among non-Māori).

**Figure 1:**
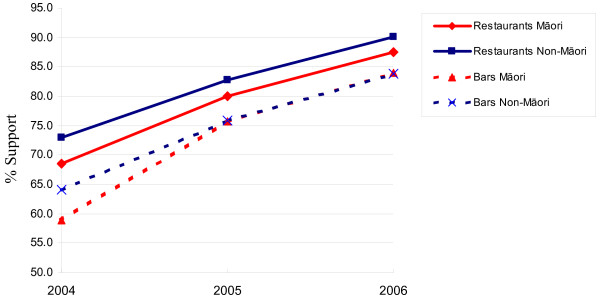
**Support for bans on smoking in restaurants and bars before and after implementation of the Smokefree Environments Amendment Act (Source: HSC Monitor Surveys 2004–6)**. Key: Support was defined as those who strongly agreed or agreed with a ban on smoking in each setting.

Finally, the level of support for a ban on smoking in indoor workplaces increased steadily between 2004 and 2006, with similar increases among Māori and non-Māori (figure 1).

### Experiences and attitudes of Māori stakeholders to the introduction and implementation of the Smokefree Environments Amendment Act

The Māori stakeholders interviewed were all largely supportive of the smokefree legislation and its implementation, though they noted that there was not universal support among Māori leaders and organisations at the time of developing and implementing the legislation. All participants stated that Māori have generally accepted this legislation and changes associated with it;

*"I'm around Māori heaps and I don't hear anyone moaning; Māori have been generally compliant, Māori are impacted equally but not separately"*.

Several believed that the SEAA had enabled a new level of discussion about smokefree homes and getting rid of tobacco smoking entirely. The call by the Māori Party MP Hone Harawera in 2006 for legislation to ban all cigarette sales was cited as evidence for this. A more recent illustration is that some Māori tobacco control advocates and practitioners have recently supported a change in the social marketing message to Māori from Auahi Kore (smokefree) to Tupeka Kore (tobacco free) [36,37].

The Māori health workers interviewed felt that sufficient information was available during implementation except for some confusion about what exactly constituted a non-enclosed outdoor area. Interviewees mostly reported that effective communication about the changes, timelines and potential impacts of the legislation was facilitated by existing networks of Māori tobacco control collectives, such as Auahi Kore groups, who met regularly at a regional and local level. In addition, interviewees reported that strong central leadership by groups such as Te Reo Mārama, a national Māori tobacco control advocacy group, and strong collaboration with advocacy organisations like ASH ensured that workers on the ground were kept well informed. Methods of dissemination mentioned included Te Reo Mārama posters, smokefree resources for use on marae, Māori radio advertising, and "*loads of hui*" (consultation and feedback meetings).

Not all of the Māori stakeholders thought that sufficient information was available to them to aid in implementing the new legislation. For example, one interviewee reported difficulties in working on implementation of the SEAA in Māori communities:

*"Māori environments and Māori speaking environments required appropriate information and people to disseminate it. So it was easy for me as a Māori and Māori speaker, but difficult for those of my colleagues who weren't. [ *There was *] not enough clarification for Māori communities; when it came to marae it was left right off the radar. The target group, as Māori, was not taken into consideration. Māori, although a minority community have a high percentage of smokers. We feel like we were targeted for change, but weren't getting the right information through to make the change."*

Most stakeholders interviewed strongly supported the legislation and cited positive impacts for Māori communities and smokers. For example, some interviewees gave anecdotal evidence of increased uptake of cessation activities within District Health Boards following implementation, and increased work for Aukati Kai Paipa (a Māori smoking cessation programme).

Some of the Māori stakeholders raised concern about the new law. For example, one stakeholder was concerned that outside spaces were becoming normalised as places for smoking. One interviewee commented: *"smokers are still role modelling to rangatahi (children and adolescents) about smoking" *(when smoking outside). Another Māori health manager was disappointed that the legislation did not apply to some indoor areas, particularly marae. However, this was in contrast to other interviewees, who felt the approach was appropriate, and that smokefree marae should be introduced by Māori communities and not imposed through legislation.

### Exposure to SHS in the workplace

In 2001 an estimated 25% of the Māori adult workforce was exposed to SHS in the workplace during worktime and 50% during work breaks [21]. This compared with estimates of 17% of all adult workers exposed to SHS in the workplace during worktime and 33% during breaks.

**Figure 2:**
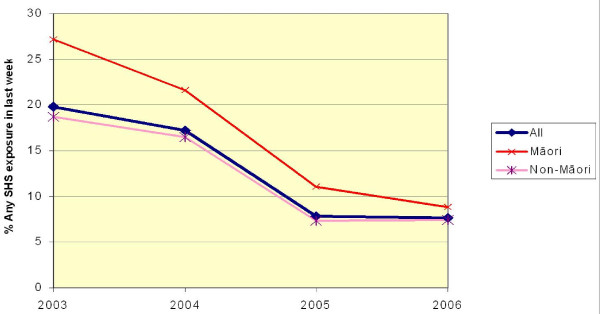
**Secondhand smoke exposure indoors at work in previous week by ethnicity, (Source: HSC Monitor Surveys 2003–6)**.

Self-reported SHS exposure from others smoking indoors at work in the previous week was reported in HSC Monitor surveys from 2003–2006. Figure 2 shows that this fell from 27.2% (95% CI 22.8% to 32.1%) in 2003 to 8.9% (95% CI 6.9% to 11.4%) in 2006 among Māori workers compared with from 18.7% (95% CI 16.3% to 21.4%) to 7.5% (95% CI 5.9% to 9.4%) among all employed adults [26]. By 2006 the difference in reported exposure between Māori and non-Māori was minimal. There were similar findings in the 2006 Tobacco Use Survey with 12.0% (95% CI 8.7% to 15.4%) of Māori and 7.6% (95% CI 6.3% to 8.8%) of non-Māori reporting SHS exposure (time period not specified) indoors in the workplace [7].

### SHS exposure in the home and other settings

Data on self-reported SHS exposure in the home was available from surveys carried out by the NRB between 1989 and 1996. These revealed that exposure was common with the proportion of adults reporting regular smoking in the home being 26% in 1989, 25% in 1991 and 28% in 1996 [19,32,33], i.e. there was little evidence of change during this period. Exposure was higher for Māori (48% in 1996) and people of Pacific Island origin (34% in 1996) households compared with European/other households (24% in 1996) [19].

The HSC Monitor surveys included questions addressing SHS exposure in the home during the previous week and whether or not there were rules on smoking in the home (table 2). Self-reported SHS exposure in the home (any reported smoking by other people in the home in the last seven days) declined most steeply in absolute terms in Māori households (households where the respondent self-identified as Māori): from 31.0% (95% CI 27.3% to 34.9%) to 16.7% (95% CI 14.4% to 19.3%) between 2003 and 2006; and in Māori households with children: from 31.6% (95% CI 27.1% to 36.5%) to 13.6% (95% CI 11.0% to 16.8%). This compared to reductions from 18.0% (95% CI 16.0% to 20.1%) to 8.4% (95% CI 7.0% to 10.0%) in non-Māori households, and from 19.8% (95% CI 16.8% to 23.2%) to 8.3% (95% CI 6.2% to 11.0%) in non-Māori households with children. [26]

**Table 2:** Proportion of households with any SHS exposure indoors in the home in the previous week and with smokefree policies in the home by ethnicity (HSC Monitor Surveys 2003–6)

	**Any SHS exposure in last week**		**No smoking policy in home**	
	Māori (95% CI)	Non-Māori (95% CI)	Māori (95% CI)	Non-Māori (95% CI)
All households				
2003	31.0 (27.3 to 34.9)	18.0 (16.0 to 20.1)	73.3 (69.5 to 76.8)	80.5 (78.4 to 82.5)
2004	23.5 (20.7 to 26.4)	15.2 (13.3 to 17.2)	74.7 (71.8 to 77.5)	80.2 (78.0 to 82.3)
2005	20.1 (17.5 to 23.0)	11.0 (9.4 to 12.8)	76.3 (73.3 to 79.1)	82.3 (80.1 to 84.2)
2006	16.7 (14.4 to 19.3)	8.4 (7.0 to 10.0)	81.3 (78.7 to 83.7)	86.1 (84.2 to 87.8)

All households with one or more children				
2003	31.6 (27.1 to 36.5)	19.8 (16.8 to 23.2)	72.7 (68.0 to 77.0)	82.0 (78.6 to 84.8)
2004	21.5 (18.1 to 25.4)	15.6 (12.7 to 18.9)	77.3 (73.4 to 80.7)	81.6 (78.2 to 84.7)
2005	20.5 (17.2 to 24.3)	9.8 (7.6 to 12.6)	76.9 (73.0 to 80.4)	86.5 (83.5 to 89.1)
2006	13.6 (11.0 to 16.8)	8.3 (6.2 to 11.0)	84.6 (81.3 to 87.3)	89.0 (86.1 to 91.3)

All households with one or more smokers				
2003	48.3 (42.3 to 54.2	40.7 (36.3 to 45.4)	57.1 (51.1 to 62.9)	60.2 (55.6 to 64.7)
2004	41.6 (37.0 to 46.4)	36.5 (32.0 to 41.2)	57.3 (52.6 to 62.0)	57.7 (52.9 to 62.4)
2005	35.4 (31.0 to 40.2)	33.9 (29.2 to 39.0)	61.0 (56.2 to 65.6)	54.6 (49.5 to 59.7)
2006	29.3 (25.2 to 33.8)	30.4 (25.3 to 35.9)	65.9 (61.3 to 70.1)	61.9 (56.2 to 67.3)

All households with one or more children and one or more smokers				
2003	47.8 (40.8 to 54.8)	38.2 (32.1 to 44.7)	58.7 (51.6 to 65.4)	65.3 (58.9 to 71.2)
2004	35.0 (29.4 to 41.0)	33.2 (26.9 to 40.2)	64.2 (58.3 to 69.7)	62.7 (55.7 to 69.3)
2005	33.5 (28.2 to 39.2)	28.3 (22.1 to 35.5)	63.0 (57.2 to 68.5)	65.6 (58.2 to 72.3)
2006	23.0 (18.4 to 28.2)	26.9 (20.2 to 34.8)	73.8 (68.5 to 78.6)	68.4 (60.4 to 75.5)

There were similar trends seen with the largest absolute and relative reductions in reported smoking in the home in Māori households which contained one or more smokers in the HSC surveys (table 2). In households with one or more smokers SHS exposure reduced from 48.3% (95% CI 42.3% to 54.2%) to 29.3% (95% CI 25.2% to 33.8%) in Māori households, and from 40.7% (95% CI 36.3% to 45.4%) to 30.4% (95% CI 25.3% to 35.9%) in non-Māori households. There was also a reduction from 47.8% (95% CI 40.8% to 54.8%) to 23.0% (95% CI 18.4% to 28.2%) in Māori households with one or more smokers and one or more children. This compared to a decline from 38.2% (95% CI 32.1% to 44.7%) to 26.9% (95% CI 20.2% to 34.8%) in non-Māori households.

The proportion of homes reported as having smokefree policies (no smoking allowed anywhere inside) also increased during this time period. For example, the proportion of households reported with smokefree policies where there were one or more smokers and one or more children living in the household, increased from 58.7% (95% CI 51.6% to 65.4%) to 73.8% (95% CI 68.5% to 78.6%) among Māori households, and from 65.3% (95% CI 58.9% to 71.2%) to 68.4% (95% CI 60.4% to 75.5%) among non-Māori households.

Reductions in self-reported SHS exposure and increases in homes with no-smoking rules were more marked in Māori compared to non-Māori households, particularly among households with one or more smokers (table 2). However, SHS exposure in the home was still statistically significantly more common (16.7% [95% CI 14.0% to 19.3%] vs 8.4% [95% CI 7.0% to 10%]), and smokefree home policies statistically significantly less common (81.3% [95% CI 78.7% to 83.7%] vs 86.1% [95% CI 84.2% to 87.8%]), among Māori compared to non-Māori households in 2006. These figures were supported by findings from the 2006 Tobacco Use Survey, where 70.3% (95% CI 66.5% to 74.1%) of Māori and 85.1% (95% CI 83.5% to 86.6%) of non-Māori respondents reported no smoking occurring in their home [7].

However, in the HSC surveys, when the analysis was restricted to households with one or more smokers, the lower rate of reported smokefree policies in homes in Māori households in 2003 had disappeared or even reversed by 2006. For example, of households in 2003 with one or more smokers and one or more children, 65.3% (95% CI 58.9% to 71.2%) of non-Māori and 58.7% (95% CI 51.6% to 65.4%) of Māori had smokefree homes, but by 2006 the figures were 68.4% (95% CI 60.4% to 75.5%) for non-Māori and 73.8% (95% CI 68.5% to 78.6%) for Māori. These differences between Māori and non-Māori were not statistically significant.

In the ASH Year 10 survey, students were asked 'do people smoke in your home?' Compared with the findings from the HSC Monitor surveys, the downward trends in exposure using this measure were less marked. Levels of reported smoking in the home remained higher among Māori children throughout 2001–2007 (table 3). There was little change in reported exposure from 2001 to 2003, but since 2003 the proportion reporting smoking in the home has declined. The degree of decline has been similar among Māori and all children. There was a particularly marked fall in reported exposure between 2003 and 2004 among Māori [38]. The fall in smoking in the home has occurred despite only minor decreases in the proportion of children reporting one or more parents smoke.

**Table 3:** Trends in proportion of Year 10 students reporting parental smoking and smoking in the home, 2001–2007 (ASH Year 10 surveys) [38]

	2001 %	2002 %	2003 %	2004 %	2005 %	2006 %	2007 %
Māori							
Parental smoker	66.0	64.8	64.3	64.1	65.2	65.1	62.8
Smoking in home	47.4	44.2	47.9	39.6	41.8	39.8	36.6

All ethnicities							
Parental smoker	40.3	39.4	40.6	41.0	39.8	39.9	39.1
Smoking in home	30.5	27.1	29.9	27.1	26.5	25.0	22.3

Another important source of SHS exposure is smoking in cars. However, there are no trend data available on SHS exposure in cars covering the period before and after implementation of the SEAA. In the 2006 NZ Tobacco Use Survey, the proportion of Māori reporting others smoking in the car was 30.1% (95% CI 26.6% to 33.6%) compared to 12.6% (95% CI 11.4% to 13.9%) among non-Māori [7].

### Smoking prevalence and smoking-related behaviours

**Figure 3:**
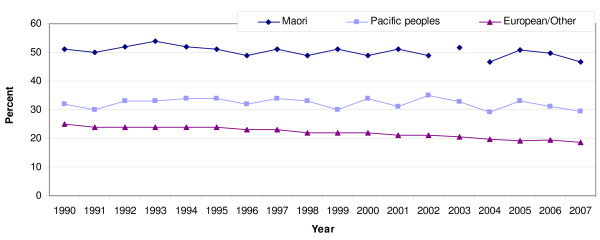
**Trends in smoking prevalence 1990 to 2007 in New Zealand by ethnicity, sexes combined. (Source: AC Nielsen surveys)**.

The impact of the SEAA on smoking prevalence among Māori is unclear. There are three main sources of data to assess trends. Annual surveys conducted by AC Nielsen suggest that the prevalence of smoking among Māori changed little between 1990 and 2005, but may have been declining slowly since 2005 (figure 3) [39].

A question on smoking status had been included in the 1996 and 2006 New Zealand national population censuses. In 1996 smoking prevalence among all adults aged 15 years and over was 23.7%. This decreased to 20.7% in the 2006 census. The figures for Māori were 43.7% in the 1996 census and 42.2% in the 2006 census [39].

Both of these data sources suggest little change in smoking prevalence among Māori over the last decade. However, data from three recent New Zealand Health Surveys gives a different picture. Age-standardised daily smoking prevalence was 25.2% (95% CI 23.7% to 26.7%) in 1996/7, 23.4% (95% CI 22.2% to 24.7%) in 2002/3 and 18.7% (95% CI 17.7% to 19.7%) in 2006/7 in all adults aged 15 years and over. The figures for Māori adults were 46.0% (95% CI 41.8% to 50.2%) in 1996/7, 47.2% (95% CI 43.8% to 50.6%) in 2002/3, and 37.6% (95% CI 35.5% to 39.7%) in 2006/7 [34]. There was no statistically significant difference in smoking prevalence among Māori between 1996/7 and 2002/3, but there was a statistically significant fall in prevalence between 2002/3 and 2006/7. These data suggest a marked decline in smoking may have occurred during the period after the SEAA was implemented.

For a six-month period after implementation of the SEAA, there was evidence of increased quitting-related behaviour, with increases in caller registrations and in the issuing of NRT vouchers through Quitline [35]. The increase was greater when a reduction in marketing of the Quitline in the period after implementation of the SEAA is taken into account, with Quitline caller registrations, per dollar of advertising directly linked to smoking cessation, at least a doubling in the six months after the law change compared to other six month periods between December 2002 and November 2005. The proportion of calls to the Quitline by Māori throughout the period from 2002–6, and including the 6 month period after implementation of the SEAA, was around 20% suggesting that the SEAA was an equal stimulus to quit among Māori and non-Māori [35].

**Figure 4:**
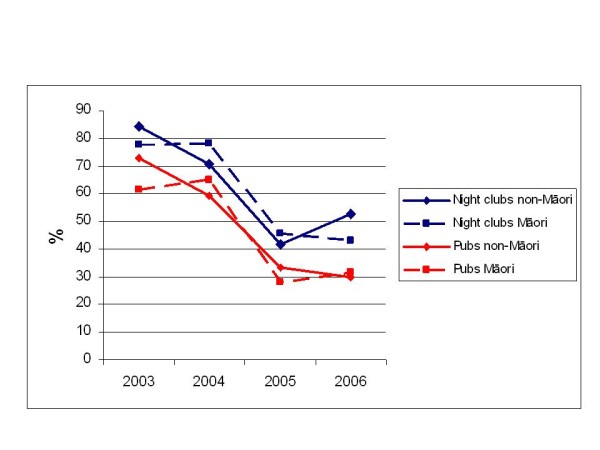
**Socially-cued smoking when visiting pubs and nightclubs 2003–6 (Source: HSC Monitor Surveys 2003–6)**.

Smokers were asked about the degree to which they smoked when visiting various hospitality industry venues in the 2003–6 HSC Monitor Surveys. Socially-cued smoking (smoking more frequently than normal in social settings) at bars and nightclubs declined substantially between 2003–4 and 2005–6. The decrease was present among Māori and non-Māori to a similar degree (figure 4). There were similar findings for socially-cued smoking in restaurants and cafés.

## Discussion

The main aim of the SEAA legislation was to reduce SHS exposure in the workplace. Surveys found that self-reported exposure to SHS in workplaces decreased greatly among Māori after implementation of the SEAA, and to a greater degree than among non-Māori. We found quantitative evidence of strong support for the smokefree legislation among Māori and non-Māori. Qualitative research with Māori stakeholders found that most were supportive of the way the legislation had been introduced and reported positively on its impacts on Māori communities. Exposure to SHS in the home reduced, and may have decreased more in Māori households, particularly those with one or more smokers. However, SHS exposure in the home remained greater in Māori households. Impact on smoking prevalence among Māori was unclear with evidence from the census suggesting little change, while the New Zealand Health Survey suggested a large fall in prevalence between 2002/3 and 2006/7. However, similar reductions in socially-cued smoking occurred among Māori and non-Māori, and Māori responded to the new law with increased calls to the national Quitline service. This is therefore one of the few documented examples that we are aware of, where a national tobacco control intervention has disproportionately benefited a relatively disadvantaged indigenous or ethnic minority population. It is therefore a demonstration of the positive effects that healthy public policy can have on health inequalities.

There is a strong imperative for carrying out such an evaluation, particularly in New Zealand given the very high rates of smoking, increased SHS exposure and smoking related harms suffered by the Māori population. Additional reasons for focusing on the impact of Māori include the lack of any cultural tradition of tobacco use among Māori, the introduction of tobacco as part of the colonisation process, and the historical lack of adequate efforts by government to protect Māori from the tobacco epidemic.

A major strength of this study is that it is the first which we are aware of to examine the impact of smokefree legislation on an indigenous population. Other strengths of the evaluation were the use of multiple data sources and a combination of qualitative and quantitative methods. Particular strengths of the data sources were that we were able to use large national surveys of adults with boosted Māori samples (Tobacco Use Survey, New Zealand Health Survey, HSC Monitor Surveys), a survey of Year 10 children (ASH Year 10 survey) with large numbers of Māori participants (over 4500 in 2006), and a series of annual surveys of adults (HSC Monitor) and year 10 children (ASH Year 10) covering the years before and after implementation of the smokefree legislation.

There were however, important limitations. The scope of the evaluation was limited and did not include assessment of the impact of smokefree schools and other educational settings. Limitations to the methods included the small sample size for the qualitative research with Māori stakeholders and a focus on interviewees involved with tobacco control or smoking cessation activities. There was no data available on impacts of the SEAA on Māori for relevant health outcomes and economic effects.

The de-normalisation of smoking in New Zealand society may have resulted in increasing social desirability bias for data based on self-reports, particularly those concerning smoking in the home and possibly smoking prevalence. For example, this may have resulted in exaggeration of the reported reductions in SHS exposure and increases in smokefree homes. The low response rates in the HSC Monitor surveys and deteriorating response rate over time for Māori may have caused bias by resulting in an under-estimate of levels and exaggerating the decreasing trend in SHS exposure in the home and workplace. The degree of this bias is uncertain. However, findings on trends for SHS exposure in the home were similar in the ASH Year 10 survey in which response rates varied less over time, and there were similar levels of SHS exposure in the home and workplace among Māori and non-Māori reported in the NZ Tobacco Use Survey, which had a response rate of 75.4% [7]. Other studies involving measuring air quality in hospitality industry settings, and salivary cotinine of bar customers suggest that the law has resulted in dramatic changes in smoking behaviour and SHS exposure, at least in these settings [40,41].

A possible general critique of our approach is that with the exception of the Māori stakeholder interviews, the data available for this evaluation did not come from Māori-led research or from studies using methods from within the Māori research paradigm. However, three of the authors of this paper are Māori and led some of the research used for this evaluation. This approach facilitated the inclusion of Māori perspectives in the research and evaluation. Additional in-depth investigation of the experience and impact of the SEAA from a Māori led perspective using kaupapa Māori research methods would be likely to add additional evidence and relevance for Māori stakeholders.

The generalisability of the findings cannot be assumed, and additional evidence is required to assess the impact of smokefree legislation with other indigenous communities, particularly where those communities have a strong cultural tradition of tobacco use, such as in North America. The existence of such cultural traditions relating to tobacco use also highlights the imperative that major public health legislation with possible wide-ranging impacts on indigenous peoples should be developed in active partnership with those peoples and not simply imposed upon them. Such an approach is highlighted within the text of the Framework Convention on Tobacco Control.

The findings of the evaluation were very positive. From a Treaty of Waitangi perspective, the evaluation suggests that the SEAA smokefree legislation was at least partially successful in engaging with and progressing health protection for Māori, as indicated by the reductions in reported exposure to SHS in the workplace and homes, and the reduction in disparities between Māori and non-Māori. An important general point is that regardless of whether or not there is a Treaty framework like in New Zealand between the Crown and Māori, there is a strong ethical obligation for reducing health inequalities between indigenous peoples and other population groups by tackling hazards such as SHS.

From a Treaty of Waitangi perspective a risk of developing and implementing the SEAA legislation was that it could be imposed on Māori by the Crown with little real participation or partnership. However, a review of the process through which the legislation was developed indicates that SEAA was initiated by a Māori member of parliament (Tukuroirangi Morgan) and was supported in Parliament by Māori Party MPs, Te Reo Mārama, and other Māori stakeholders. Given the importance of respecting indigenous rights in policy formulation and implementation, we recommended that future evaluations of tobacco control legislation affecting indigenous peoples include process evaluation. Such evaluations should investigate how indigenous people were engaged in developing and implementing the legislation.

An intriguing finding from the HSC Monitor surveys data was that by 2006, although SHS exposure in the home was almost twice as common among Māori compared with non-Māori respondents, there was no difference in reported SHS exposure in the home between Māori and non-Māori respondents living in households with one or more smokers, and slightly less SHS exposure reported by Māori participants living in households with children and one or more smokers (table 2). A similar pattern was present for the presence of no-smoking rules in the home. This suggests that by 2006 the higher SHS exposure in the home and lower proportion of smokefree homes reported by Māori participants may be attributable simply to higher smoking prevalence in Māori households, and no longer partly due to higher rates of smoking inside by smokers in Māori households, as was the case in 2003.

An important issue that was identified through the evaluation was whether or not it was appropriate that the legislation did not stipulate that marae should be smokefree (although where marae were also educational institutions, a place of work or had a liquor licence the Smokefree legislation would apply). There was some disagreement among the Māori stakeholders interviewed about this. There was some debate in Parliament about this during 2003, and also some media commentary. Two main issues were raised. The first was whether government legislation on Māori settings impinges on self-determination rights, as marae are under the jurisdiction of local iwi or hapu. The second issue was whether banning smoking in Māori settings is discriminatory as they are domestic settings, not unlike private homes which were not covered by the legislation. This is a little studied issue, however, our impression is that, in practice this was largely an issue for local discussion at individual marae, and many marae are now smokefree. However, this would be a useful area for additional research for example to quantify the numbers and proportion of smokefree marae and to explore qualitatively the issues and processes where smokefree marae policies are discussed or implemented.

## Conclusion

The findings of our evaluation of the impact of the 2003 New Zealand smokefree legislation on an indigenous people were very positive, though there were some concerns about the process of implementation among a minority of Māori stakeholders. We believe this evaluation highlights the need for similar research to be conducted to assess the impact of public health legislation and policies on indigenous peoples, especially those who live within countries with a colonial history. In addition, research investigating the impact of public health legislation with other population groups such as immigrant ethnic minority populations and socio-economically disadvantaged populations would be helpful. Rigorous evaluation of the impact of major public health legislation on indigenous peoples should be incorporated into the evaluation plans and appropriate data collection and studies planned in advance of the implementation of the legislation or policy change.

## Competing interests

The evaluation and some of the primary data collection was funded by the New Zealand Ministry of Health. Other primary research studies were funded by a variety of agencies detailed in the relevant publications. The views expressed are those of the authors and do not necessarily represent those of the Ministry of Health.

AW has no competing interests to declare.

RE, HG, GT, NW, MG, have previously undertaken contract work for not-for-profit organisations involved in tobacco control

MG is on the Champix advisory board for Pfizer, and has been on similar boards for GSK Zyban in the past, and carried out contract work for Novartis training Aukati Kai Paipa in use of NRT many years ago.

## Authors' contributions

RE led the drafting of this paper and the design and implementation of the evaluation project on which it was based.

HG, AW, NW, GT and MW participated in developing and implementing the evaluation and in preparing the evaluation report. All provided details comments on drafts of this paper, and have approved the final version.

The stakeholder study was led by HG and GT. Additional analysis of the HSC Monitor Survey data was led by RE. The bars air quality study was led by NW and RE. Analysis of Quitline data was led by NW.

## References

[B1] Tobacco Advisory Group of the Royal College of Physicians (2005). Going smoke-free: the medical case for clean air in the home, at work and in public places.

[B2] Ministry of Health (2003). Smoke-free Environments Amendment Act 2003.

[B3] Ministry of Health (1990). Smoke-free Environments Act 1990.

[B4] Edwards R, Thomson G, Wilson N, Waa A, Bullen C, O'Dea D, Gifford H, Glover M, Laugesen M, Woodward A (2008). After the smoke has cleared: Evaluation of the impact of a new national smokefree law in New Zealand. Tob Control.

[B5] Ministry of Health (2005). Tobacco Facts 2005.

[B6] Statistics New Zealand (2007). Demographic Trends (2006) – reference report.

[B7] Ministry of Health (2007). New Zealand Tobacco Use Survey.

[B8] Laugesen M, Clements M (1998). Cigarette Smoking Mortality among Māori 1954–2028.

[B9] Blakely T, Fawcett J, Hunt D, Wilson N (2006). What is the contribution of smoking and socio-economic position to ethnic inequalities in mortality in New Zealand?. The Lancet.

[B10] Wilson N, Blakely T, Tobias M (2006). What potential has tobacco control for reducing health inequalities? The New Zealand situation. Int J Equity Health.

[B11] Ministry of Health (2002). He Korowai Oranga (Māori Health Strategy).

[B12] Durie MH (1994). Whaiora: Māori health development.

[B13] Royal Commission on Social Policy (1988). The April Report: Report to the Royal Commission on Social Policy. Wellington.

[B14] Broughton J (1996). Puffing up a storm: "Kapai te torori".

[B15] Pool I (1991). Te Iwi Māori a New Zealand population past, present, and projected.

[B16] Harris A (2004). Hikoi: Forty years of Māori protest.

[B17] Māori killers (Te Reo Mārama). http://www.resist.co.nz/.

[B18] Apārangi Tautoko Auahi Kore (2003). National Māori Tobacco Control Strategy, 2003–2007.

[B19] National Research Bureau (1996). Environmental tobacco smoke survey.

[B20] National Research Bureau (1999). Attitudes towards environmental tobacco smoke.

[B21] National Research Bureau (2001). Exposure to second-hand cigarette smoke.

[B22] Ministry of Health (2000). The New Zealand Health Strategy.

[B23] Ministry of Health (2003). The New Zealand Cancer Control Strategy.

[B24] Ministry of Health (2005). Clearing the smoke: a five-year plan for tobacco control in New Zealand (2004–2009).

[B25] Health Committee of New Zealand Parliament (2003). Smoke-free Environments (Enhanced Protection) Amendment Bill, Member's Bill: as reported from the Health Committee.

[B26] Edwards R, Bullen C, O'Dea D, Gifford H, Glover M, Laugesen M, Thomson G, Waa A, Wilson N, Woodward A (2006). After the Smoke has Cleared: Evaluation of the Impact of a New Smokefree Law.

[B27] Gifford H (2003). He Arorangi Whakamua: reducing the uptake of tobacco in Ngati Hauiti rangatahi. PhD Thesis.

[B28] MacDonald G, Starr G, Schooley M, Klimowski K, Turner K (2001). Introduction to program evaluation for comprehensive tobacco control programs.

[B29] Haw SJ, Gruer L, Amos A, Currie C, Fischbacher C, Fong G (2006). Legislation on smoking in enclosed public places in Scotland: how will we evaluate the impact?. J Public Health.

[B30] Waa A, McGough S (2006). Reducing exposure to second hand smoke: changes associated with the implementation of the amended New Zealand Smoke-free Environments Act 1990: 2003–2006.

[B31] Scragg R (2006). Report of the 1999–2005 National Year 10 smoking surveys.

[B32] National Research Bureau (1989). Monitor of heart health behaviour of adult New Zealanders.

[B33] National Research Bureau (1991). Monitor of heart health behaviour of adult New Zealanders.

[B34] Ministry of Health (2008). A portrait of health: key results of the 2006/7 New Zealand Health Survey.

[B35] Wilson N, Sertsou G, Edwards R, Thomson G, Grigg M, Li J (2007). A new national smokefree law increased calls to a national quitline. BMC Public Health.

[B36] Te Reo Mārama, Smokefree Coalition, Action on Smoking and Health (2007). Tobacco Control – The way forward: "Aotearoa/New Zealand Tobacco Free Tupeka Kore!".

[B37] Northland District Health Board (2008). Tupeka Kore Te Tai Tokerau: Tobacco Free Northland 2008 to 2011 (draft).

[B38] Paynter J (2008). National Year 10 ASH Snapshot Survey, 1999–2007: Trends in tobacco use by students aged 14–15 years.

[B39] Ministry of Health (2008). Tobacco Trends 2007: a brief update on monitoring indicators.

[B40] Lea R, Fowles J, Fernando D, Christophersen A, Woodward A, Dickson S, Hosking M, Berezowski R (2006). Secondhand tobacco smoke exposure in New Zealand bars.

[B41] Wilson N, Edwards R, Maher A, Nathe J, Jalali R (2007). Air quality inside hospitality settings after a national smokefree law in New Zealand. BMC Public Health.

